# Single-cell RNA sequencing reveals heterogeneity among AT2 epithelial cells in the lung adenocarcinoma microenvironment

**DOI:** 10.3389/fimmu.2025.1735459

**Published:** 2025-12-03

**Authors:** Zimeng Cao, Xuefeng Bai, Jing Li, Jinru Cai, Gang Cao, Yuncai Xiao, Wei Zhou

**Affiliations:** 1 National Key Laboratory of Agricultural Microbiology, Huazhong Agricultural University, Wuhan, China; 2 College of Life Science Technology, Huazhong Agricultural University, Wuhan, China; 3 College of Biomedicine and Health, Huazhong Agricultural University, Wuhan, China; 4 Baotou Cancer Hospital, Baotou, China; 5 College of Veterinary Medicine, Huazhong Agricultural University, Wuhan, China; 6 Shenzhen Institute of Advanced Technology, Chinese Academy of Sciences, Shenzhen, China

**Keywords:** single-cell RNA sequencing, lung adenocarcinoma, AT2 epithelial cells, tumor microenvironment, TEAD1, CADM1

## Abstract

**Introduction:**

Lung cancer is a highly heterogeneous disease, and the tumor microenvironment (TME) characteristics are closely related to disease progression and treatment outcomes. We elucidated the cell type-specific transcriptome landscape of cancer cells and the effect of the TME on lung adenocarcinoma (LUAD).

**Methods:**

Single-cell RNA sequencing of the LUAD tissues and matched adjacent normal tissues of three patients in the early-stage via histopathological and immunohistochemical confirmation.

**Results:**

The results revealed the landscape of 68,579 cells in LUAD microenvironment, and highly heterogeneous AT2 cells are crucial source of lung epithelial cell carcinogenesis. Genes *KRT81*, *SPP1*, *PCDH7*, *SLC2A1*, and *TET1* were significantly upregulated in tumor tissues and associated with poor prognosis and survival, providing insights for exploring lung cancer biomarkers in future studies. Trajectory analysis identified *ERBB4*, *SEMA4A*, *GCNT2* and *SOX4* as key factors in AT2 cells that may promote cell proliferation, migration, or epithelial-mesenchymal transition (EMT) during the progression of lung adenocarcinoma (LUAD). We elucidated the transcription factor–target gene regulatory network involving NKX2-1 and TEAD1 in malignant tumor cells derived from AT2 cells. The findings indicated that malignant AT2 cells regulate communication between epithelial and immune cells in the TME by predicted FN1-CD44 and CADM1-CADM1 ligand-receptor interactions, which ultimately suppresses the host immune response.

**Discussion:**

This comprehensive single-cell analysis increases our understanding of AT2 cells molecular and dynamics of metastatic lung cancer. In summary, single-cell RNA profiling of LUAD offers valuable prognostic insights based on AT2 cell types and identifies potential biomarkers for therapeutic responses, aiding the future development of LUAD treatment strategies.

## Highlights

- A total of 68,579 cells from LUAD patients revealed heterogeneous transcriptomic profiles of AT2 tumor cells and facilitated the identification of cancer cell subtypes that deviated from the normal differentiation trajectory.
- KRT81, SPP1, PCDH7, SLC2A1, and TET1 are associated with poor prognosis and survival outcomes, providing novel insights for exploring lung cancer biomarkers.
- NKX2–1 and TEAD1 gene interaction networks in AT2 cell clusters are strongly associated with LUAD progression.
- Malignant AT2 epithelial cells evade immune surveillance by regulating the predicted CADM1–CADM1 and FN1–CD44 signaling pathways.

## Introduction

Among all cancer types, lung cancer is the most common malignancy worldwide and exhibits the highest morbidity and mortality rates (1), accounting for 1.796 million deaths (18%) annually (2). Non-small cell lung cancer (NSCLC) accounts for more than 85% of lung cancer cases (3), with lung adenocarcinoma (LUAD) accounting for approximately 40–50% of all NSCLC cases (4, 5). To date, numerous studies have focused on exploring T cells for their effective antitumor immunotherapy applications (6). Despite the success of these therapeutics against LUAD tumors, the response rates are relatively low. For instance, only approximately 20–25% of patients respond to immuno-oncology drugs (7, 8). The low drug response rate is likely due to the complex network of cell–cell interactions present in the tumor microenvironment (TME) (9). Cancer immunotherapy relies heavily on a comprehensive understanding of the immune landscape of the TME. Diverse groups of predictors of the responses to therapy have been identified in the TME, including genomic features, transcriptomic signatures, and epigenetic modifications, which also contribute to the heterogeneity in the clinical response across cancer subtypes and orchestrate either beneficial or adverse outcomes for tumor progression (10, 11). Recent advances in immuno-oncology have demonstrated that the characteristics of both cancer cells and the TME can guide precision medicine (12). As immunotherapeutic strategies become more prevalent and complex, understanding the mechanisms underlying malignant immune cell remodeling in the TME of LUAD and identifying potential intervention targets to increase the effectiveness of immunotherapy are necessary.

Single-cell RNA sequencing (scRNA-seq) approaches are increasingly being used to characterize both the abundance and functional state of tumor-associated cell types and have provided unprecedented details on the heterogeneity in the cellular population. ScRNA-seq facilitates comprehensive transcriptome profiling at the single-cell resolution with an unbiased catalog of cellular diversity and is a promising tool for extensively investigating immune heterogeneity in the TME (13, 14). The epithelial–mesenchymal transition (EMT) plays important roles in determining the invasion, metastasis, and chemoresistance of solid tumors (15, 16). Epithelial cells are involved in various normal and cancer cell states, and their diversity among cancer cells is closely linked to the LUAD microenvironment (17). The current paradigm of LUAD etiology posits that alveolar epithelial type II (AT2) cells are the primary malignant cells (18). Therefore, we performed scRNA-seq on the AT2 subcluster of epithelial cells in our study.

We performed an scRNA-seq analysis of 68,579 high-quality cells collected from the tumor tissues of 3 patients with LUAD and matched adjacent normal tissues to identify clinically relevant microenvironmental and cancer features. We comprehensively characterized the immune microenvironment in both normal and LUAD tissues, establishing a single-cell transcriptome atlas for all major subtypes of LUAD. We observed a diverse TME landscape in LUAD, characterized by a significant reduction in the interaction network strength between malignant cells and immune cells, especially the FN1–SDC4, FN1–CD44, HBEGF–ERBB4, EREG–ERBB4, and CADM1–CADM1 interactions. Pseudotime and regulatory network analyses provided the first evidence that the interaction between the *PATJ* gene and TEAD1 could serve as a potential therapeutic immunotherapeutic target and novel specific marker for LUAD.

The complex interactions among malignant cells and immune cells, along with the comprehensive profiling of the LUAD microenvironment, may help reveal novel clinically relevant tumor subtypes and immunotherapy targets on the basis of microenvironmental interaction features. This work highlights that malignant cells in LUAD tumor tissues exhibit an enhanced immune evasion capacity.

## Results

### Single-cell RNA sequencing reveals the transcriptional landscape of the lung adenocarcinoma microenvironment

**Figure 1:**
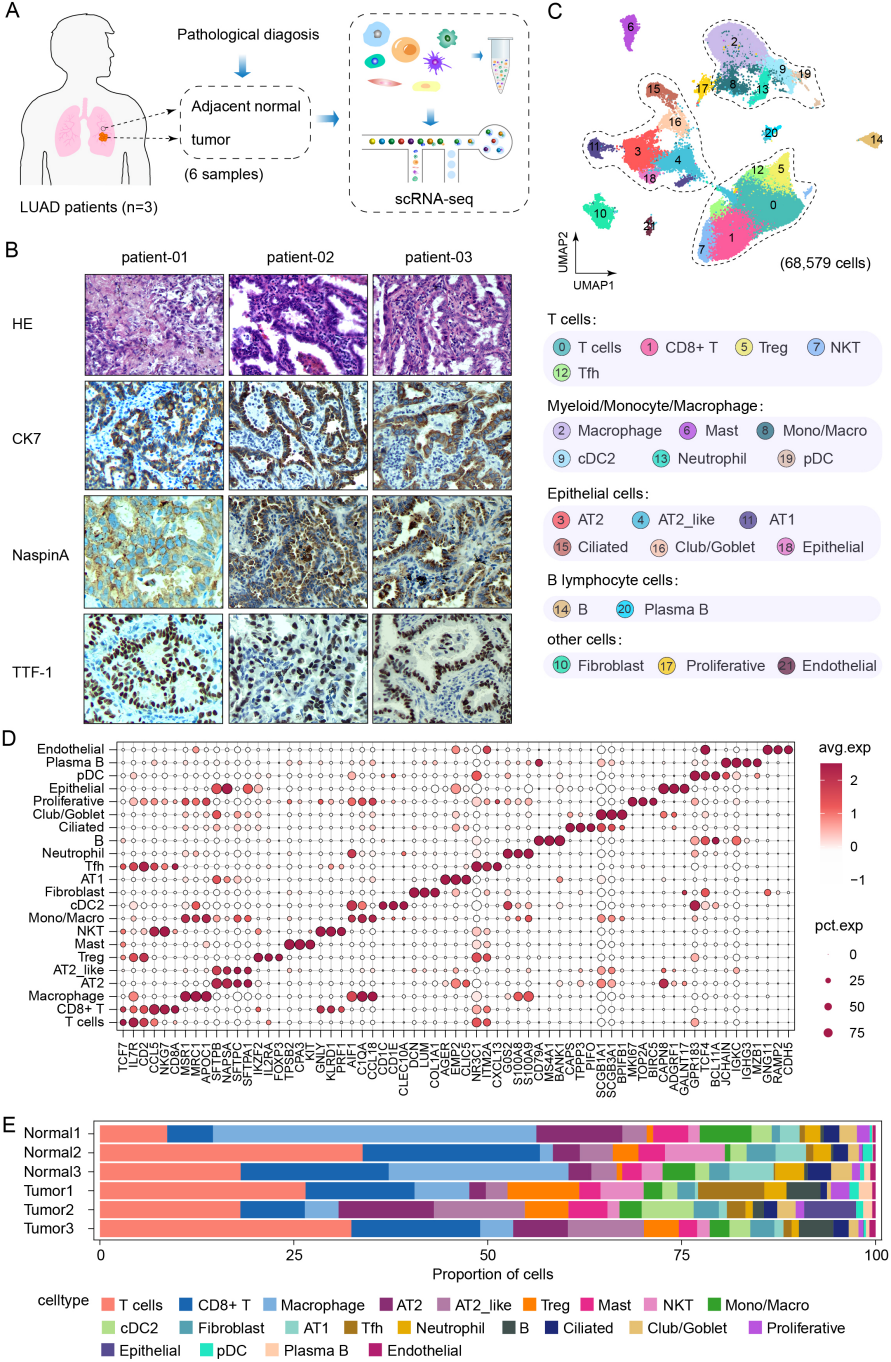
Single-cell RNA sequencing reveals the transcriptional landscape of lung adenocarcinoma. **(A)** Schematic of the study design and samples information. **(B)** H&E staining and Immunohistochemical classification of target CK7, NaspinA and TTF-1 in lung tissues from LUAD patients. **(C)** UMAP plot of 68,579 high quality cells were annotated to twenty-two cell types, including tumor tissues (n=3) and adjacent normal lung tissues (n=3). **(D)** Dot plot of the expression of canonical marker genes for each cell type. The dot size is proportional to the fraction of cells expressing the specific gene. The color intensity corresponds to the relative expression of each specific marker gene. **(E)** Bar plot of the relative percentages of each cell type in each tissue specimen.

A total of 6 freshly resected lung samples were collected from 3 patients with the same histologic subtype of LUAD (CK7^+^, Naspin A^+^, and TTF-1^+^) for a biomarker analysis (**Figure 1B**), along with 3 adjacent normal lung tissues from a distal region within the same lobe, which served as controls (**Figure 1A**). We performed droplet-based scRNA-seq, and following the implementation of quality control, a total of 68,579 high-quality cells were retained for analysis. Cells were identified according to the expression of key marker genes and assigned to 22 major types, including T cells (CD2^+^), CD8^+^ T cells (CD8A^+^), macrophages (MRC1^+^), AT2 cells (SFTPB^+^), AT2_like cells (SFTPC^+^), regulatory T cells (IL2RA^+^), mast cells (CPA3^+^), natural killer cells (PRF1^+^), monocytes (C1QA^+^), cDC2 cells (CD1C^+^), fibroblasts (DCN^+^), AT1 cells (AGER^+^), follicular helper T cells (NR3C1^+^), neutrophils (G0S2^+^), B cells (CD79A^+^), ciliated cells (CAPS^+^), club/goblet cells (SCGB1A1^+^), proliferative cells (MKI67^+^), epithelial cells (CAPN8^+^), plasmacytoid dendritic cells (pDCs) (GPR183^+^), plasma B cells (JCHAIN^+^), and endothelial cells (GNG11^+^), (**Figures 1C, D**). Moreover, the relative proportions of each cell cluster in each tumor tissue and normal tissue sample from the 3 LUAD patients were evaluated (**Figure 1E**). The proportions of cell clusters among the tumor and normal samples from each patient were highly heterogeneous. The degree of infiltration differed across the major cell types, and adaptive immune responses were activated to varying extents, reflecting differences in LUAD progression. Notably, a previous report revealed that immune evasion starts as early as the preneoplastic stage, at which time the enrichment of T and B lymphocytes and NK cells decreases (19, 20), which differs from our results. Moreover, given the findings of previous studies suggesting that AT2 cells are the origin of LUAD (20, 21), we focused primarily on the epithelial cell cluster for further analysis.

### Landscape and heterogeneity in malignant epithelial cells

**Figure 2:**
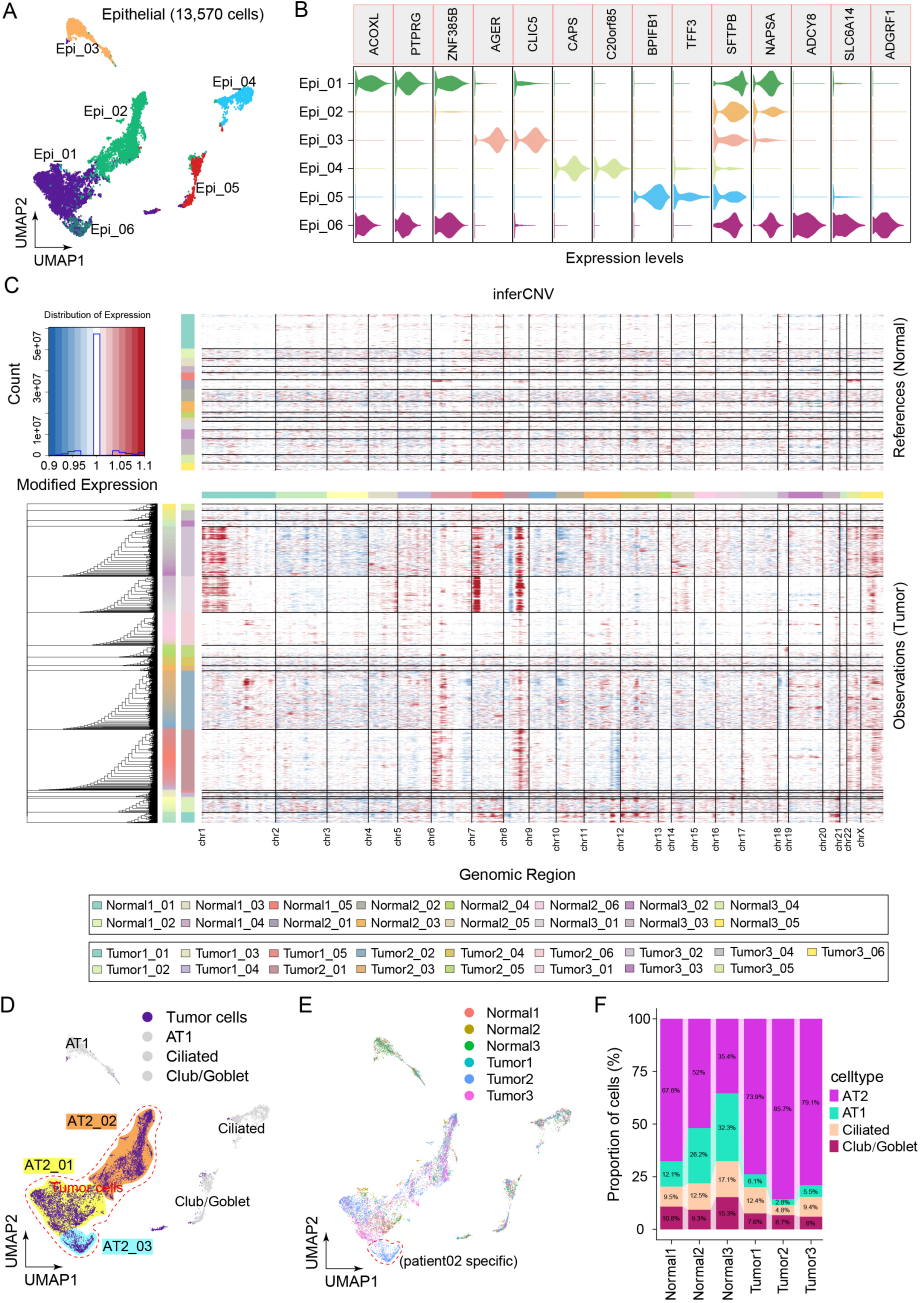
Landscape of epithelial cell heterogeneity in the LUAD microenvironment. **(A)** Clusters display of 13,570 epithelial cells from all the samples named Epi_01, Epi_02, Epi_03, Epi_04, Epi_05, Epi_06. **(B)** Violin plots show the distribution of specific marker gene state scores in each cluster. **(C)** The heatmap displayed large-scale CNVs in the 13,570 epithelial cells from tumor tissues and adjacent normal tissues. Red represents a high CNV, and blue represents a low CNV in tumor samples compared with normal samples. **(D)** Four main clusters were identified via UMAP analysis, and AT2 cells were renamed tumor cells. **(E)** UMAP plots of epithelial cells colored to display the patient source heterogeneity. **(F)** Bar plot showing the relative percentages of AT2, AT1, ciliated and club/goblet cell types across the six clinical samples.

We reclustered the epithelial cells via uniform manifold approximation and projection (UMAP) dimensionality reduction to characterize the specific immune-related behaviors of epithelial cells (**Figure 2A**). Within the epithelial cell cluster, a total of 13,570 cells were classified as Epi_01 (ACOXL^+^), Epi_02 (SFTPB^+^), Epi_03 (AGER^+^), Epi_04 (CAPS^+^), Epi_05 (BPIFB1^+^), and Epi_06 (ADCY8^+^) (**Figure 2B**). InferCNV was employed to distinguish between tumor cells and normal cells among the epithelial cells on the basis of large-scale somatic copy number variation (CNV) events (**Figure 2C**). Chromosomal amplification and deletion events were mapped to each chromosomal position of the epithelial cells (**Figure 2C**). The results revealed large-scale chromosomal CNVs for Epi_01, Epi_02, and Epi_06. Although only tumor sample 2 contained cluster Epi_06, the marker genes of Epi_06 were nearly identical to those of Epi_01 and Epi_02. Moreover, Epi_01, Epi_02, and Epi_06 all belong to the AT2 cell population subset and were renamed AT2_01, AT2_02, and AT2_03, respectively. Therefore, AT2_01, AT2_02, and AT2_03 were considered clusters of malignant tumor cells (**Figure 2D**). Next, we focused on the composition ratios of the AT2_01, AT2_02, and AT2_03 clusters in the normal and tumor tissue samples (**Figure 2E**). The proportions of major cell types in each clinical sample are shown in **Figure 2F**. A greater percentage of AT2 clusters was observed in the tumor tissue samples than in the samples of adjacent normal tissue. The relative abundance of malignant cells was greatest in tumor tissues; additionally, malignant cells were observed in adjacent normal tissues, indicating that the patients may have experienced metastasis of varying severity levels (**Figure 2F**). These results suggested that malignant cells originate from AT2 cells and that the heterogeneity in tumor cells of the AT2 lineage may result in differing proliferative and invasive characteristics among patients.

### Gene expression profiles of AT2 cells reveal malignant cell heterogeneity

**Figure 3:**
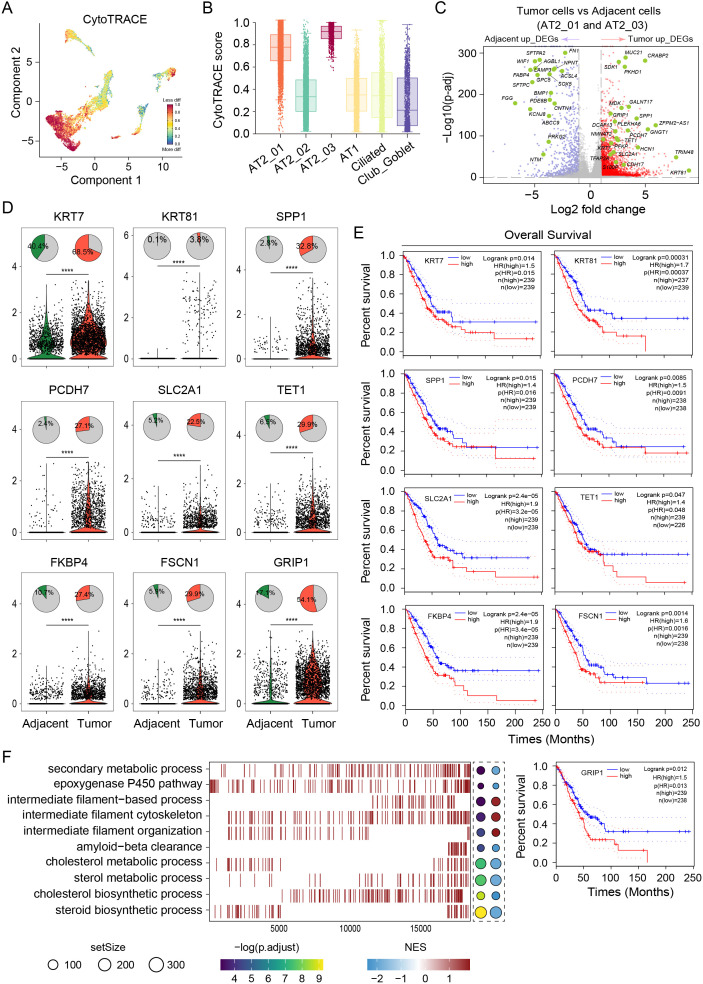
Gene expression profiles of AT2 cells reveal malignant cell heterogeneity. **(A)** UMAP of malignant tumor cells based on the differentiation state inferred from the CytoTRACE analysis. Red color represents the less differentiation. **(B)** Comparisons of the CytoTRACE scores among the AT2_01, AT2_02, AT2_03, AT1, Ciliated and Club_Goblet clusters. **(C)** Volcano plot of differentially expressed genes in both AT2_01 and AT2_03 clusters between the tumor tissue group and adjacent normal tissue group. Red dots represent significantly upregulated genes in the tumor group, and blue dots represent significantly upregulated genes in the adjacent normal tissue group. **(D)** Top panel, distribution of the proportions of cells in which target genes were expressed among epithelial cells from LUAD tumor tissues and adjacent normal tissues. Bottom panel, violin plots comparing gene expression between epithelial cells from LUAD tumor tissues (red) and adjacent normal tissues (green). *p* values of fixed effects were calculated using Student’s t test. * *p* < 0.05, ** *p* < 0.01, *** *p* < 0.001, and **** *p* < 0.0001. **(E)** TCGA database was used to predict survival and revealed significantly shorter OS in patients with high expression of the target genes *KRT7, KRT81, SPP1, PCDH7, SLC2A1, TET1, FKBP4, FSCN1*, and *GRIP1*. **(F)** GO enrichment analysis of the differentially expressed genes in **(C)**. The dot size is proportional to the gene set expressing the specific genes. The left panel color intensity corresponds to the adjusted *p* value of the enriched GO pathway. the right panel color intensity corresponds to the enrichment score of the gene set.

The differentiation states of malignant cells exhibited high interpatient heterogeneity. Cells from the AT2_01 and AT2_03 clusters were poorly differentiated, as indicated by their highest CytoTRACE scores (**Figures 3A, B**). We investigated the differentially expressed gene in LUAD tumor cell types from clusters AT2_01 and AT2_03 for comparison with adjacent normal tissues to elucidate how tumor cells can affect their biological characteristics. 2,460 genes was upregulated and 710 genes was downregulated. Most of the upregulated genes displayed cancer subtype-specific features and functions, as shown in the volcano plot (**Figure 3C**). These upregulated genes (*KRT7, KRT81, SPP1, PCDH7, SLC2A1, TET1, FKBP4, FSCN1*, and *GRIP1*) were expressed at higher levels in tumor tissues than in adjacent normal tissues, which indicated a poor prognosis and shorter survival of LUAD patients according to TCGA predictions (http://gepia.cancer-pku.cn/) (**Figures 3D, E**). The results of GO enrichment analysis of the DEGs (**Figure 3F**) revealed that the DEGs in LUAD tumor tissues were enriched mainly in the negative regulation of secondary metabolic processes, negative regulation of epoxygenase P450 pathways, positive regulation of intermediate filament-based processes, positive regulation of the intermediate filament cytoskeleton, positive regulation of intermediate filament organization, negative regulation of amyloid-beta clearance, negative regulation of cholesterol metabolic processes, negative regulation of sterol metabolic processes, negative regulation of cholesterol biosynthetic processes and negative regulation of steroid biosynthetic processes. These results suggest that tumor cells play a major role in promoting proliferation and invasion during LUAD progression. In conclusion, malignant cells exhibited reduced stemness and different expression levels of genes among patients, which explains the variations in heterogeneity, complexity, and metastatic capacity among individuals with LUAD. In-depth research into the immune microenvironment can aid in the exploration of additional novel target genes for LUAD therapy.

### Developmental trajectory of epithelial subtypes in LUAD

**Figure 4:**
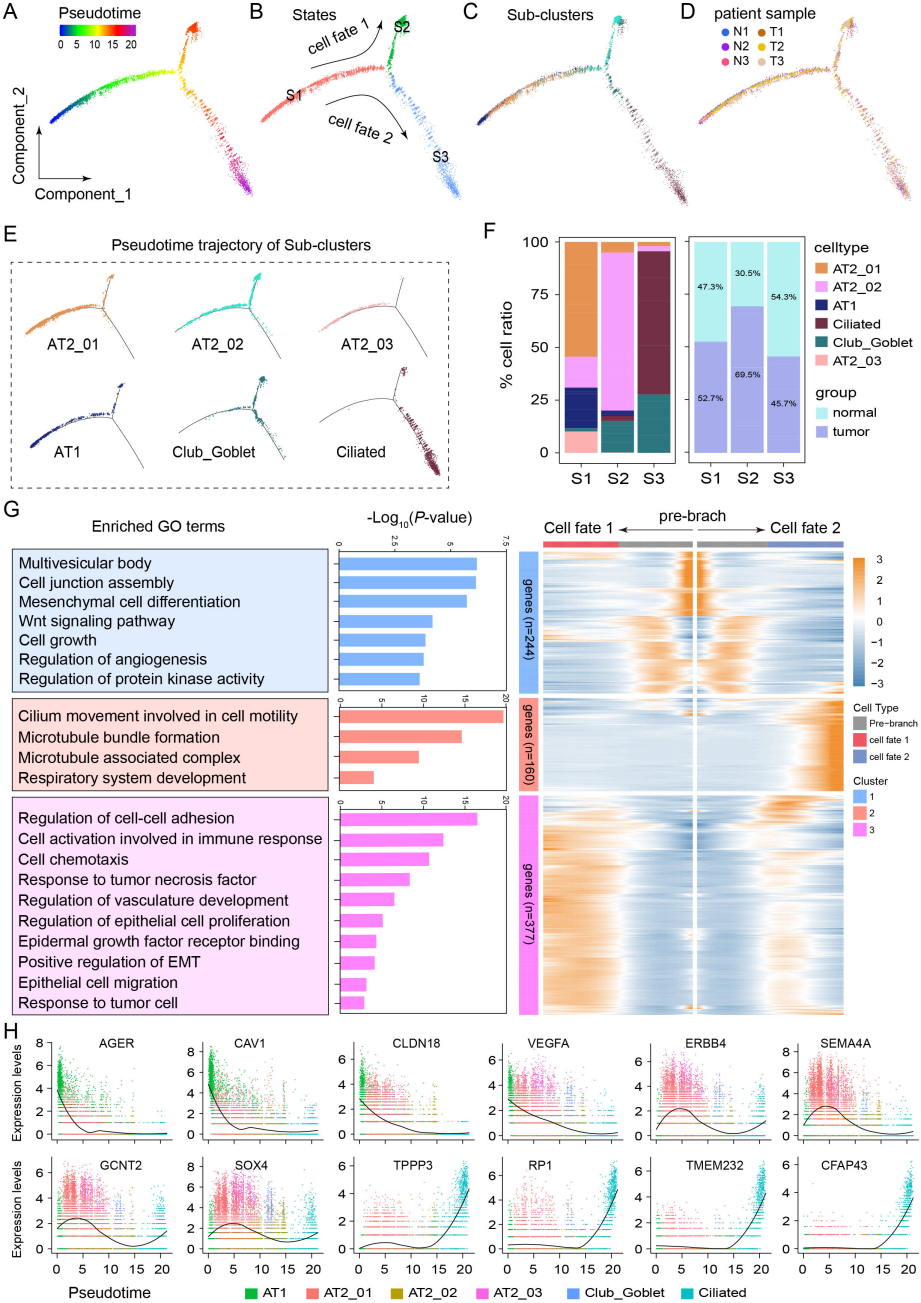
Developmental trajectory of epithelial cell subtypes to malignant cells in the LUAD microenvironment. **(A)** Monocle2 pseudotime trajectory analysis of all epithelial cells. **(B)** The trajectory analysis of epithelial cells revealed 3 cellular states, including cell fate 1 (S1 to S2) and cell fate 2 (S1 to S3). **(C)** Developmental trajectory of epithelial cell sub-clusters in different cell states and different patient samples **(D)**. **(E)** Pseudotime trajectory of the cell states of AT2_01, AT2_02, AT2_03, AT1, Club_Goblet and Ciliated cells. **(F)** Average proportion of each epithelial cell subtype in different states or groups. **(G)** Heatmap of 781 differentially expressed genes arranged in three pseudotime patterns. The enriched Gene Ontology terms revealed biological functions or signaling pathways involved in regulating LUAD progression. **(H)** Representative expression levels of crucial genes in different epithelial cell developmental trajectories.

Monocle2 was used for pseudotime analysis to identify the key molecular events governing the cell fate transition during the progression from normal cells to cancer cells. The epithelial cells were ordered along a putative developmental trajectory (**Figure 4A**). A pseudotime line with bifurcation containing two cell fates at the most differentiated timeline was obtained, and the pseudotimeline was divided into the following 3 states: S1, S2, and S3 (**Figure 4B**). Further developmental trajectory analyses revealed a similar trajectory between different subclusters and patients (**Figures 4C, D**). Clusters AT2_01, AT2_02, AT2_03, AT1, Club/Goblet, and Ciliated were distributed in different stages of S1, S2, and S3 with specific profiles (**Figure 4E**). The AT2_01 (≥ 50%) was enriched mainly in S1, the AT2_02 (≥ 75%) was enriched mainly in S2, and the Ciliated (≥ 60%) and Club/Goblet (≥ 25%) were enriched mainly in S3. In S1 and S2, tumor cells were more abundant than normal cells were. However, S3 contained more normal cells (**Figure 4F**). Subsequently, a branched expression analysis was performed to identify branch-dependent genes across the above three states. A total of 781 genes that regulated the cell differentiation process from S1 (pre-branch) to either S2 (cell fate 1) or S3 (cell fate 2) were identified. The results of Gene Ontology (GO) analysis revealed the dynamic transcriptional programs associated with each state. Pathway enrichment analysis of all the DEGs in S1 revealed that this state was involved in the regulation of cell-related pathways, such as the multivesicular body, cell junction assembly, mesenchymal cell differentiation, Wnt signaling, cell growth, regulation of angiogenesis, and protein kinase activity pathways. GO analysis of all the DEGs in S2 revealed that this state is involved in the regulation of carcinogenesis-related pathways, such as regulation of the cell–cell adhesion pathway, activation of cells involved in the immune response pathway, the cellular chemotaxis pathway, the response to tumor necrosis factor pathway, regulation of the vasculature development pathway, regulation of the epithelial cell proliferation pathway, the epidermal growth factor receptor binding pathway, positive regulation of the EMT pathway, the epithelial cell migration pathway, and the response to tumor cells pathway. GO analysis of all the DEGs in S3 revealed that these DEGs were enriched mainly in terms related to cilium movement, including the cell motility pathway, the microtubule bundle formation pathway, the microtubule-associated complex pathway, and the respiratory system development pathway (**Figure 4G**). During the developmental trajectory, the expression levels of *TPPP3*, *RP1*, *TMEM232*, and *CFAP43* increased at the end stage, whereas the expression levels of *AGER*, *CAV1*, *CLDN18*, and *VEGFA* decreased; moreover, *ERBB4*, *SEMA4A*, *GCNT2* and *SOX4* were expressed at the early stage (**Figure 4H**). These results reveal the critical roles of these genes and signaling pathways in shaping the two distinct pathways driving the malignant fate of AT2 cells.

### NKX2–1 and TEAD1 gene interaction networks in AT2 clusters are strongly associated with LUAD progression

**Figure 5:**
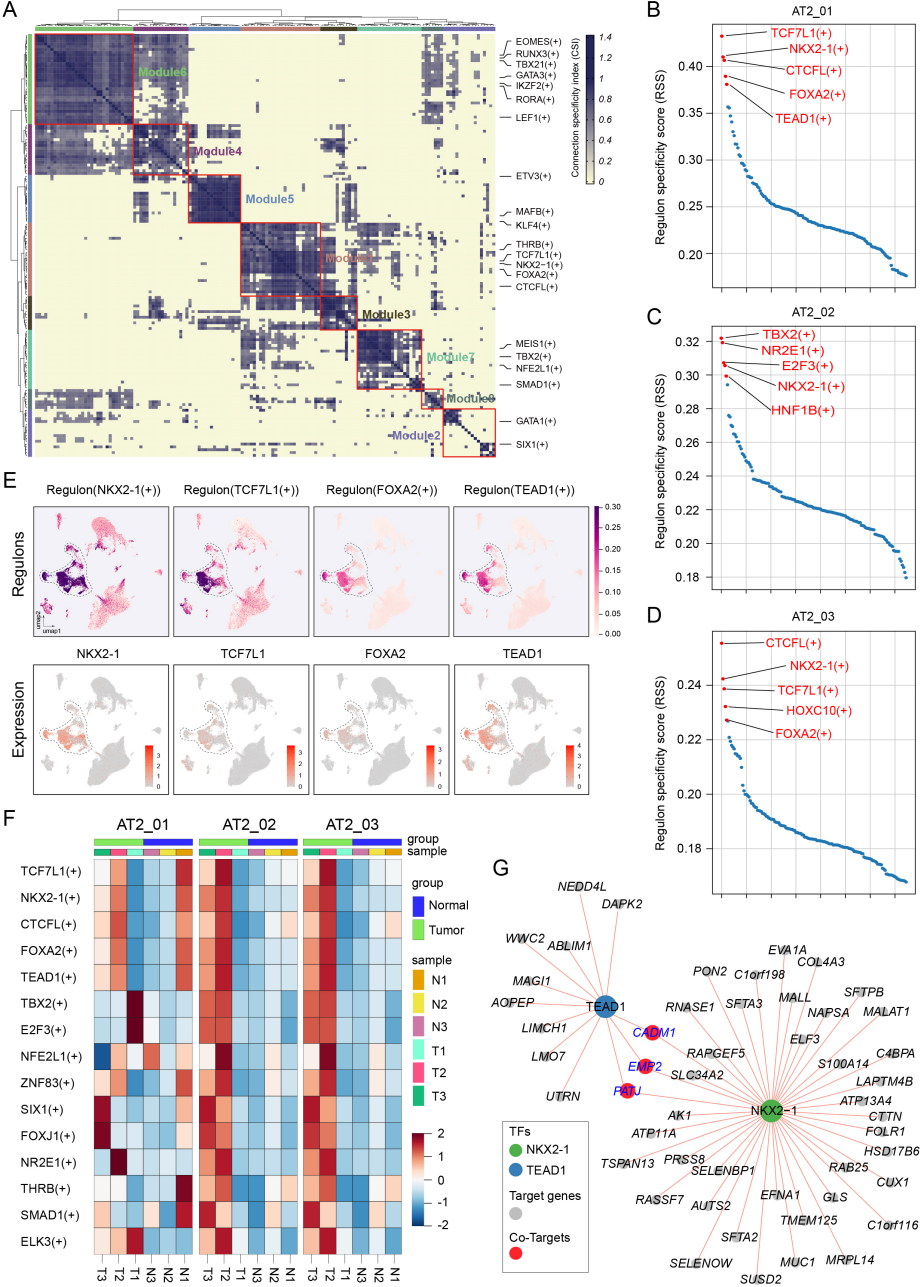
The NKX2–1 and TEAD1 regulon networks in AT2 cells were strongly associated with LUAD progression. **(A)** Regulon modules identified using the regulon CSI matrix. **(B)** The top 5 representative regulons displayed in the AT2_01 cluster, AT2_02 cluster **(C)**, and AT2_03 cluster **(D)** according to the regulon specificity score. **(E)** Regulons NKX2-1(+), TCF7L1(+), FOXA2(+) and TEAD1(+) identified via UMAP (upper panel) and gene expression levels were displayed via UMAP plot (lower panel). **(F)** Heatmap of the crucial regulons highly expressed in AT2 clusters estimated in different samples and patients using SCENIC package. **(G)** Gene interaction network analysis of the transcription factors TEAD1 and NKX2–1 and their target genes. The length of the line segment denotes the co-expression relationship score.

The single-cell regulatory network inference and clustering (SCENIC) technique is a novel computational method used to construct regulatory networks and identify different cell states based on an analysis of scRNA-seq data (22). The SCENIC method is applied to identify significantly active regulons in the dataset. By applying a modified SCENIC approach, a total of 150 significant regulons were identified. Strikingly, these 150 regulons were organized into 8 major modules on the basis of their expression patterns (**Figure 5A**). For each module, the average activity scores of every cluster and subcluster were analyzed; subsequently, we focused on the subclusters of the epithelial cell cluster in module 1. Certain genes encoding key transcription factors were specifically expressed in different clusters, as follows: *TCF7L1*, *NKX2-1* (TTF-1), *CTCFL*, *FOXA2*, and *TEAD1* were specifically expressed in the AT2_01 cluster (**Figure 5B**); *TBX2*, *NR2E1*, *E2F3* and *NKX2-1* were specifically expressed in the AT2_02 cluster (**Figure 5C**); and *CTCFL*, *NKX2-1*, *TCF7L1* and *FOXA2* were expressed in the AT2_03 cluster (**Figure 5D**). Next, we mapped the expression of the transcription factor-encoding genes *NKX2-1*, *TCF7L1*, *FOXA2*, and *TEAD1* and their target genes in every cell via UMAP (**Figure 5E**). SCENIC analysis revealed crucial transcription factors in the tumor tissues of the AT2_01, AT2_02, and AT2_03 clusters, and the heatmap revealed high heterogeneity in the expression of transcription factors in the LUAD samples from the different patients (**Figure 5F**). The transcription factor–target network of TEAD1 and NKX2–1 was highly activated in the epithelial cell cluster and played a profound role in driving transcriptional regulation during LUAD progression. Network analyses were performed to identify the genes downstream of TEAD1 and NKX2-1, and their functions were analyzed through a gene interaction network. *CADM1*, *EMP2*, and *PATJ* were potential target genes of the transcription factors TEAD1 and NKX2-1 (**Figure 5G**).

### Malignant epithelial cells evade immune surveillance by suppressing the CADM1 signaling pathway

**Figure 6:**
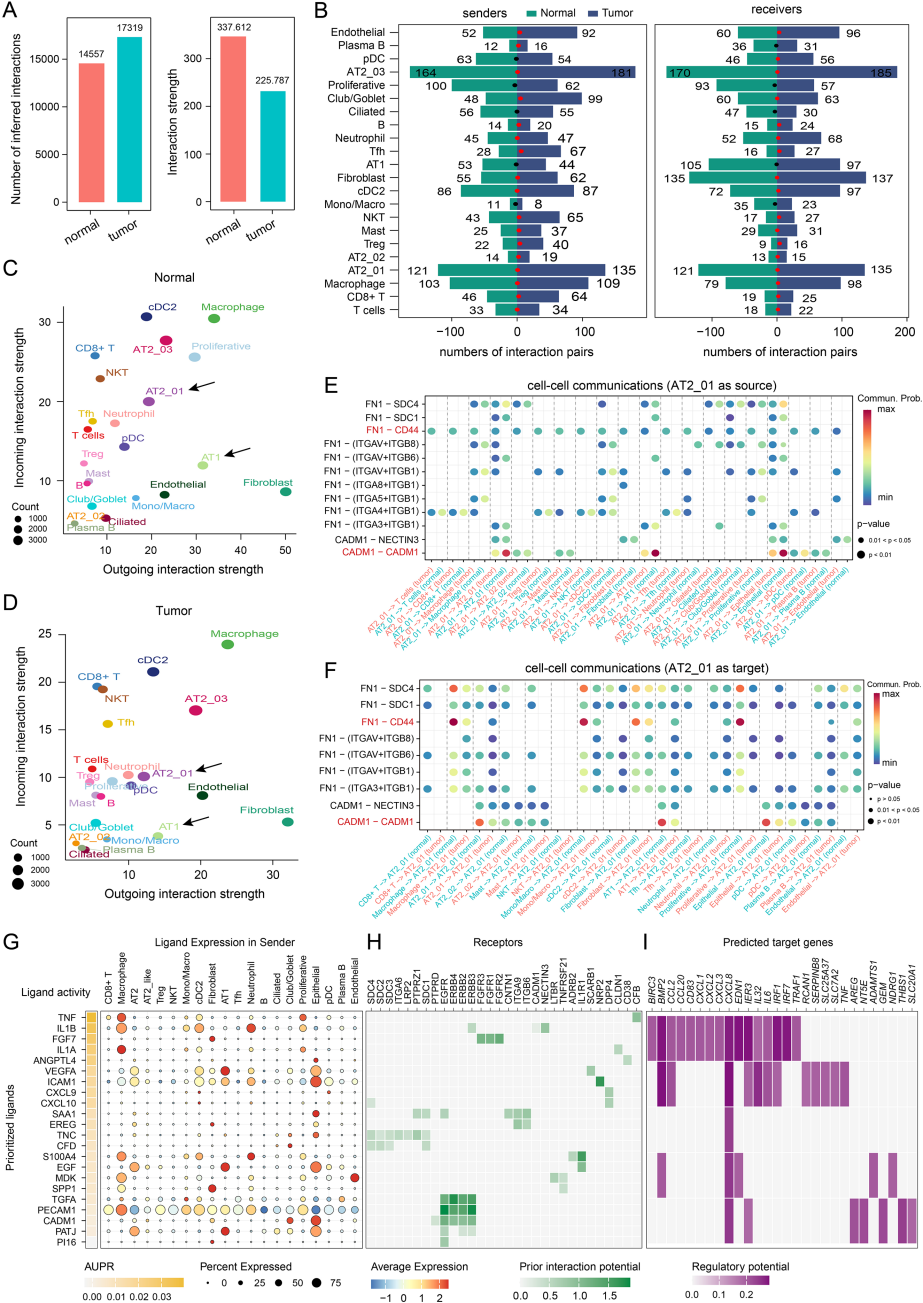
Analysis of malignant epithelial cell–cell communication and ligand–receptor interactions. **(A)** Bar plot of the inferred numbers of interactions and interaction strengths in the LUAD tumor tissue group and adjacent normal tissue group. **(B)** Bar chart showing the number of significant ligand–receptor interaction pairs in each cell type. The ligand (left panel) and receptor (right panel) pairs were calculated separately for each cell subtype. The dots represent the ratio of number of significant ligand–receptor pairs between tumor and normal tissues, with ratios greater than 1 shown in red and less than 1 shown in black. **(C)** Analysis of the incoming and outgoing interaction strengths of each cellular subtype in tumor and adjacent normal tissues **(D)**. Prediction analysis of cell-cell communications by using CellChat v2 with AT2_01 cluster as source **(E)** and AT2_01 cluster as target **(F)**. The color intensity represents the probability of putative ligand–receptor interactions, and the dot size corresponds to the *p* value. **(G)** Prioritized ligands inferred to regulate ligand activity in each cell subtype according to the NicheNet analysis. Dot plots showing the expressed cell proportion (dot size) and expression level (color intensity) of the ligands used as senders in each cell subtype. **(H)** Receptor predictions showing potential interactions in each cell subtype ordered by ligand–receptor pairs. **(I)** Heatmap showing the predicted target genes involved in the regulation of predicted ligand–receptor interactions. The statistics analysis of predicted ligand–receptor interactions were performed by NicheNet v2 package in R 4.4.0 software.

Next, the roles of all cell clusters in shaping the ligand–receptor crosstalk between LUAD tumor and adjacent normal tissues were evaluated. The statistical results revealed that the number of inferred interactions in tumor tissues was significantly greater than that in normal tissues. However, the magnitude of the interactions in tumor tissues was smaller than that of the interactions in normal tissues (**Figure 6A**). As a method to investigate the interactions that occur in each subcluster, CellChat v2 was used to calculate the numbers and strength of the cell–cell interactions. Compared with that in adjacent normal lung tissues, the number of interactions in most subclusters was significantly greater, except in the proliferative, ciliated, AT1, and mono/macro clusters (**Figure 6B**). However, outgoing interaction strength levels in malignant cell subclusters (such as AT1, AT2_01, AT2_02, and AT2_03) were relatively lower than those in adjacent normal tissues (**Figures 6C, D**). Afterward, the differences in the potential crosstalk signaling pathways based on ligand–receptor interactions were identified. Considering the CNV event and malignant cell proportion results obtained from LUAD tumor tissues, the cell subtypes that interacted with the AT2_01 subcluster were our main focus. Notably, compared with normal tissues, tumor tissues exhibited high levels of ligand–receptor interactions, especially putative FN1–CD44 interactions in the FN1 signaling pathway and putative CADM1-CADM1 interactions in the CADM signaling pathway, which may be able to recruit immune cells into the TME and inhibit tumor progression (**Figures 6E, F**). Moreover, as revealed by the NicheNet analysis, the majority of cells exhibited high ligand activity and expression of the *TNF*, *IL1B*, *IL1A*, *ICAM1*, *CADM1*, and *PATJ* genes. In addition, an IL1B-encoding protein bound to receptors encoded by *ERBB3, CADM1*, and *NECTIN3*; an IL1A-encoding protein bound to the receptor encoded by *CLDN1*; an ICAM1-encoding protein bound to the receptor encoded by *NRP2*; and ligands encoded by CADM1 interacted with receptors encoded by *PTPRD, EGFR, ERBB4, ERBB2*, and *ERBB3*. In particular, *CADM1* was identified as a cotarget gene (**Figure 5G**), and *ERBB4* was the key gene involved in the two cell fates determined from the pseudotime line (**Figure 4H**). Therefore, the regulation of the CADM1–ERBB4 pathway plays important roles in driving tumor cell malignant transformation and LUAD progression. A total of 28 predicted targets were involved in cell proliferation and apoptosis (e.g., collagens encoded by *BIRC3*, *BMP2*, *IL32*, *IRF1*, *TRAF1*, *AREG*, and *THBS1*), as well as inflammation-related processes (e.g., *CXCL1*, *CXCL2, CXCL8*, and *IL6*) (**Figure 6E**).

Taken together, these results demonstrated that malignant epithelial cells in the TME exhibited an enhanced immune evasion capacity through the cell–cell communication via the predicted FN1-CD44 and CADM1 signaling pathway.

## Discussion

In this study, we established a compendium of the high-resolution single-cell immune landscape in LUAD tissues and identified significant differences in immune microenvironmental signatures between LUAD tumor tissues and adjacent normal tissues. A total of 22 different cell types were identified in the LUAD microenvironment, and the characteristics of these tumor-associated subsets were confirmed. Notably, the AT2_03 cluster was specific to patient_02. Moreover, this study revealed that not all patients exhibited immune evasion by reducing the enrichment of T and B lymphocytes and NK cells, illustrating the heterogeneity in the TME among patients. This study revealed that the suppressed cell–cell interaction network of malignant and immune cells potentially contributes to the formation of an immunosuppressive microenvironment.

Despite progress in surveillance and treatment strategies and the increase in overall survival rates for LUAD patients, the clinical outcomes of LUAD remain poor because of the high incidence of early recurrence, even after surgical resection. Characterization of the TME at single-cell resolution can provide insights into potential novel therapeutic targets (12). By applying scRNA-seq to LUAD tumor tissues and adjacent normal tissues, we identified several target genes whose high expression levels predict a poor prognosis; these genes could be targeted in future studies for LUAD drug therapies. Pseudotime analysis projected malignant cells onto two different branches. Certain target genes (e.g., *ERBB4*, *SEMA4A*, *GCNT2* and *SOX4)* play critical roles in shaping two pathways related to the malignant fate of AT2 cells, which are inseparable from tumor differentiation and migration, suggesting that these genes may be novel therapeutic targets for further studies (23). Our data provide in-depth insights into cancer immunology and are essential resources for drug discovery. More comprehensive characterization of the microenvironment will likely provide even deeper insights for patient stratification and drug development.

Previous studies have revealed that the expression of the *NKX homeobox-1* (*NKX2-1*) gene, also referred to as *thyroid transcription factor-1* (*TTF-1*), is a specific biomarker of lung carcinomas (24). Therefore, *CADM1* and *EMP2* are strongly associated with the LUAD prognosis (25, 26). By integrating our regulatory network and interaction network data, we showed that the transcription factors TEAD1 and NKX2–1 were highly activated in the malignant cluster and that target genes of both transcription factors, such as *TEAD1, CADM1, EMP2, and PATJ*, could serve as candidate biomarkers for the prognosis and treatment of LUAD in the future. However, the putative FN1–CD44 and CADM1–CADM1 interactions in the tumor center could reveal the mechanisms underlying the malignant effects of epithelial cells on LUAD (27–29). These cellular interactions can trigger the recruitment of immune cells into the TME and inhibit tumor progression. Malignant cells avoid host immunity from the tumor immune microenvironment and evade host immune surveillance, potentially leading to poor prognosis and rapid progression in LUAD patients. Our study found that gene *PATJ* may play an important role in shaping the occurrence and progression of cancer and that its regulator TEAD1 could serve as a specific marker for LUAD diagnosis.

scRNA-seq provides both a comprehensive census of microenvironmental cell types and an in-depth functional characterization of their transcriptional profiles (30). However, the current techniques cannot be used to distinguish tumor tissues from the TME, and the use of resections directly aided by human eyes can result in the incorrect evaluation of the boundaries between the two types of tissues, leading to inaccurate identification and statistical analyses. Furthermore, in-depth *in vivo* and *in vitro* experiments are needed to validate our findings and assess the actual clinical value of our study.

In summary, our findings suggest the prognostic importance of TME patterns, which could aid in therapeutic decision-making. However, differences in the immune microenvironment among patients with the same molecular cancer type remain to be determined. The extent to which LUAD cancer cells shape their microenvironment and to what extent the microenvironment, in turn, influences LUAD cancer cells remain unknown. In the future, assessing the immune environment of tumors and identifying various subtypes of immune responses (31) are key to ultimately increase patient survival rates.

## Materials and methods

### Patients and sample collection

All the samples in this study were obtained from patients who underwent standard-of-care surgical resection of early-stage LUAD (I–IIIA) with histopathological and immunohistochemical confirmation and were evaluated at Baotou Cancer Hospital. Tumor tissues and adjacent normal tissues were obtained from 3 treatment-naive Chinese patients and subjected to single-cell sequencing analysis. Based on the location of the tumor within the sample, incisions were made in one direction at defined collection sites, and adjacent normal tissue was the tumor-distant normal parenchyma 0.5 cm from the tumor edge. Fresh samples were stored in GEXSCOPE tissue preservation solution (Singleron Biotechnologies) at 2–8°C.

### Tissue dissociation and preparation of single-cell suspensions

Fresh tumor tissues and adjacent normal tissues were washed with iced Hanks balanced salt solution (HBSS) three times and maintained on ice for immediate processing. The samples were transferred to a new dish on ice and minced into small pieces, followed by enzymatic digestion. Briefly, small pieces of the samples were placed in 20 mL of a digestion solution and incubated for 40 min with shaking at 37 °C. After digestion, the cells were passed through a 40-μm sterile strainer to remove impurities. The cells were subsequently centrifuged at 300 × *g* for 5 min at 4 °C, after which the cell pellets were washed twice with PBS. Freshly prepared cell suspensions were counted with a Luna cell counter to determine the cell concentration and viability. The cell suspensions were prepared for single-cell 3’-transcriptome library construction.

### Single-cell RNA sequencing

MobiCube High-throughput Single-Cell 3’-Transcriptome Set v2.1 (PN-S050200301) and the MobiNova-100 microfluidic platform were used for scRNA-seq. The single-cell suspension was adjusted to an appropriate concentration (700–1200 cells/μL) and immediately loaded onto a chip for microdroplet formation on the MobiNova-100 platform. Reverse transcription, cDNA amplification, and DNA library construction were performed according to MobiDrop protocols (Zhejiang, China), and scRNA-seq libraries were constructed and sequenced at OE Biotech Co., Ltd. (Shanghai, China) with an Illumina NovaSeq platform according to the manufacturer’s instructions.

### Single-cell RNA sequencing data processing and quality control

The FASTQ files of raw sequencing data were processed and aligned to the human reference genome (GRCh38) using MobiVision software (version 3.0), with unique molecular identifier (UMI) counts summarized for each barcode. Each output, which constituted a raw unique molecular identifier (UMI) count matrix, was subsequently transformed into a Seurat object using the Seurat package in R v5.0.1 (30). Cells quality control was performed with nFeature_RNA > 200 & nFeature_RNA < 7500 & nCount_RNA < 60000 & percent.MT < 15 & percent.RB < 30 conditions. After that, cells were regarded as high-quality cells and prepared for subsequent analyses (32). Next, each filtered gene expression matrix was normalized and log-transformed using Seurat’s NormalizeData function; i.e., the raw gene counts for each cell were divided by the total counts for that cell. Finally, the identification of 3000 highly variable genes of each Seurat object was performed by running Seurat’s FindVariableFeatures function using the following parameters: x, selection.method = “vst,” and nfeatures = 3000.

### Detection and removal of doublets and cell cycle effects

The CellCycleScoring function was used to assess and remove the effects of the cell cycle from all cells. The expression levels and proportions of canonical lineage-related marker genes in each identified cluster were carefully reviewed. Clusters coexpressing discrepant lineage markers were identified and removed. Doublets or multiplets were also identified using the doublet detection algorithm in DoubletFinder software.

### Cell clustering and annotation

The data were scaled with the top 3000 most variable genes with the FindVariableFeatures function in the Seurat package in R v5 for principal component analysis (PCA), and FindNeighbors in Seurat was employed to identify nearest neighbors for graph clustering on the basis of the PCs. FindClusters was used to determine cell subtypes and visualize cells with the UMAP algorithm. The RunUMAP function was applied with the parameters reduction = harmony and dims = 1:40 to exclude other dimensions typically correlated with sequencing depth. Genes from a specific cluster were compared with those in all other clusters using the FindAllMarkers function in Seurat to identify marker genes for each cluster. Marker genes were identified as those whose average expression was 0.5-fold higher in one particular cluster than in any other cluster. The clusters were then annotated manually according to the highly expressed marker genes.

### Differential gene expression analysis

The FindAllMarkers function in Seurat was applied to identify genes that were differentially expressed between tumor tissues and adjacent normal tissues via the use of the following parameters: min.pct = 0.25, logFC.threshold = 1, and only.pos = T. The nonparametric Wilcoxon rank-sum test was performed to determine *p* values for the comparisons and the adjusted *p* values based on the Bonferroni correction for all genes. Volcano maps or heatmaps were used to visualize the DEGs on the basis of their expression after log transformation and scaling.

### Cell–cell communication analysis

CellPhoneDB (v.2.0) was used to investigate the ligand–receptor interactions between tumor cells and immunocytes on the basis of the scRNA-seq data (33). The cell–cell contact ligand–receptor interactions in CellPhoneDB, which were categorized according to the CellChat database, were employed as inputs (34). Only the significant immune checkpoint-related interaction pairs (p < 0.05) are displayed (35). The interaction weight score was the sum of the gene expression counts of all the significant ligand–receptor pairs between two cell types.

### Inference of the pseudotime trajectory and pathway enrichment analysis

We performed pseudotime and trajectory analyses using the Monocle2 package in R to elucidate the pseudotime and lineage trajectories of immune cells in the LUAD microenvironment. Next, we adopted the functions slingPseudotime and slingCurveWeights to deduce the pseudotime and lineage trajectories, respectively. Marker genes for each cluster were defined as those whose logFC > 1 and min.pct > 0.25 according to the FindAllMarkers function. GO pathway enrichment analysis was subsequently performed on these marker genes, and a network of enriched pathways was generated.

### SCENIC analysis

The SCENIC method involves the following three main steps: coexpression analysis, target gene motif enrichment analysis, and evaluation of regulon activity. The SCENIC analysis was performed as previously described (23) using the pySCENIC (version 0.11.2) and hg38_refseq_r80_10kb_up_and_down_tss species databases for RcisTarget, GRNboost, and AUCell scoring. The input matrix was the normalized expression matrix that was extracted from the Seurat object.

### H&E staining and immunohistochemical staining

Tissue sections (3–5 μm thick) were prepared from 4% formalin-fixed, paraffin-embedded (FFPE) samples. For H&E staining, the sections were stained for 8 min with an acidic hematoxylin staining solution and for 2.5 min with an eosin staining solution (ServiceBio, China) according to standard procedures. For immunohistochemical staining, tissue sections were incubated with citric acid repair solution buffer for 30 min at 100 °C and subsequently with primary antibodies at a dilution of 1:100 overnight; then, the sections were washed with poly(butylene succinate-co-terephthalate) (PBST) 3 times. Secondary antibodies were added to the sections and incubated for 1 h at room temperature. Images were captured using an Olympus IX73 light microscope (Olympus, Japan).

### Statistics

All statistical analyses were performed using R software (version 4.4.0) and QC process of raw matrix files were performed using Seurat package (version 5.0.1). Random seeds were fixed (123) for reproducibility. *p* < 0.05 was considered statistically significant. Statistics annotation format: significance is indicated by asterisks with thresholds defined in each legend (* *p* < 0.05, ** *p* < 0.01, *** *p* < 0.001).

## Data Availability

The datasets presented in this study can be found in online repositories. The names of the repository/repositories and accession number(s) can be found below: https://ngdc.cncb.ac.cn/gsa-human, HRA011312.
